# An Implementation Strategy to Develop Sustainable Surveillance Activities Through Adoption of a Target Operating Model

**DOI:** 10.3389/fpubh.2022.871114

**Published:** 2022-04-06

**Authors:** Natalie K. Lee, Miles A. Stewart, Jessica S. Dymond, Sheri L. Lewis

**Affiliations:** Johns Hopkins University Applied Physics Laboratory, Laurel, MD, United States

**Keywords:** surveillance, public health, capacity building, low-middle-income countries, pathogen genomics, data architecture

## Abstract

The increasing threat of emerging and re-emerging pathogens calls for a shared vision toward developing and maintaining global surveillance mechanisms to enable rapid characterization of pathogens, a foundational requirement for effective outbreak response. Efforts establishing new surveillance programs in low- and middle-income countries (LMICs) have repeatedly led to siloed systems that prove unsustainable or ineffective due to narrowly focused approaches, competing priorities, or lack of resourcing. Barriers inherent to LMICs, such as resource limitations, workforce strain, unreliable supply chains, and lack of enduring champions exacerbate implementation and sustainability challenges. In order to improve adoption and endurance of new surveillance programs, more effective design and implementation of programs is needed to adequately reflect stakeholder needs and simultaneously support population-level disease monitoring and clinical decision-making across a range of chronic and acute health issues. At the heart of this cross-sectorial integration between clinical care and public health initiatives are emerging technologies and data modalities, including sequencing data. In this prospective, we propose an implementation strategy for genomics-based surveillance initiatives in LMICs founded on the use of a target operating model. Adoption of a target operating model for the design and implementation of genomic surveillance programs will ensure programs are agile, relevant, and unified across diverse stakeholder communities, thereby increasing their overall impact and sustainability.

## Introduction

The emergence of the COVID-19 pandemic has clearly demonstrated the importance of an expansive and dynamic global health surveillance network; however, as outlined in the 2021 Global Health Security Index, no country has adequate preparedness and control measures to prevent and respond to emerging infectious diseases and surveillance mechanisms proved insufficent to meet the public and global health demands (1, 2). Public health surveillance systems, such as the WHO's Global Influenza Surveillance and Response System, have proven the value of pre-existing health surveillance infrastructure that can pivot to address emerging needs during health crises (3, 4). Despite decades of progress, these surveillance programs have proven insufficiently scalable or far-reaching to meet the demands of the pandemic (5).

Advanced sequencing techniques have proven invaluable in our collective efforts to detect SARS-CoV-2 and characterize its evolution throughout the pandemic (6, 7). While there has been rapid innovation in sequencing technologies and data analytics that enable high-throughput and cost-effective pathogen discovery and detection (8, 9), the full impact of these advances in infectious disease surveillance has yet to be realized. Prior to the COVID-19 pandemic, only 39% of low-income countries had contributed influenza virus genome sequences to public databases, in comparison to 78% of upper-middle and high-income countries, indicating a significant gap in operational genomics-based surveillance capacity (10). Across Africa, nearly three quarters of the roughly 200 available sequencers are located in just five countries, the vast majority of which are situated in privately owned genomic sequencing institutions (11, 12). For LMICs that do have pathogen genomics capacity, these efforts are often siloed (i.e., operating within a single sector and/or for a specific disease), with data analysis and interpretation performed outside the host country or even the continent (13). Health surveillance systems are inherently complex, and implementation programs for pathogen genomics in LMICs are confronted with additional roadblocks such as sparse infrastructure and limited resources (14–16). Efforts to expand these capabilities during the pandemic have faced compounding challenges, competing with other resource-intensive priorities in medical and laboratory settings which require a steady flow of supplies (17, 18).

Promising initiatives aim to dramatically improve genomic surveillance capacity. USAID has invested over one billion dollars in capacity development in LMICs (19), and implementation partners such as National Institutes of Health (NIH) Fogarty International Center continue to strengthen capacity in partner nations (20). A noteworthy recent example is the Africa Pathogen Genomics Initiative. This $100 million four-year collaboration between the Africa Centers for Disease Control and Prevention (CDC) Institute of Pathogen Genomics, US CDC, Bill & Melinda Gates Foundation, and industry leaders, aims to create an integrated pan-African disease surveillance and laboratory network, including a major genomic surveillance capability, to bolster public health responses to infectious disease (11, 12). These promising efforts provide strong models for multi-national collaboration and capacity development; however, such extraordinary investment cannot be expected at a global scale, and, once established, these systems must be self-sustaining and enduring to be effective in bolstering national and global preparedness.

There is clear acknowledgment by the global health community of the need to build surveillance systems that are no longer reactionary to individual outbreak events, but are flexible and sustainable, serving as ready resources in future infectious disease outbreaks. These objectives require re-envisioning how surveillance programs are designed and implemented, maximizing opportunities for long-term impact while minimizing requirements for ongoing investment. Critical factors for consideration include feasibility of epidemiological and genomic data integration into large-scale systems, relevance to decision-makers from the individual to national level, modular technologies enabling pathogen-agnostic detection, and cost-effective workflows to ensure resource requirements are matched to local and national contexts. To meet these challenges, the design of surveillance programs must be carefully evaluated and prioritized, particularly in LMICs where resources and infrastructure are constrained.

The framing of this problem space in the corporate world, described as optimizing resource investment to achieve strategic priorities, is traditionally defined as a target operating model (TOM). This approach relies on development of a roadmap to formulate and execute strategic objectives to reach a desired end state. A TOM also provides stakeholders a high order visualization and a shared understanding of the organizational components that comprise the operational workflow and how they unite to support desired objectives. This vantage allows for a shared mission and promotes consistency, critical in building a unified genomic surveillance system given the numerous stakeholders and range of needs to be supported by the workflow. Systems engineering best practices also provide means to model and deconstruct complex systems which are inherent in the operationalization of genomic surveillance, facilitating the identification of gaps in the TOM. Here, we propose a TOM for the design, development, and implementation of enduring genomic surveillance programs in LMICs.

## A Target Operating Model for Implementation of Genomics-Based Surveillance Programs

**Figure 1:**
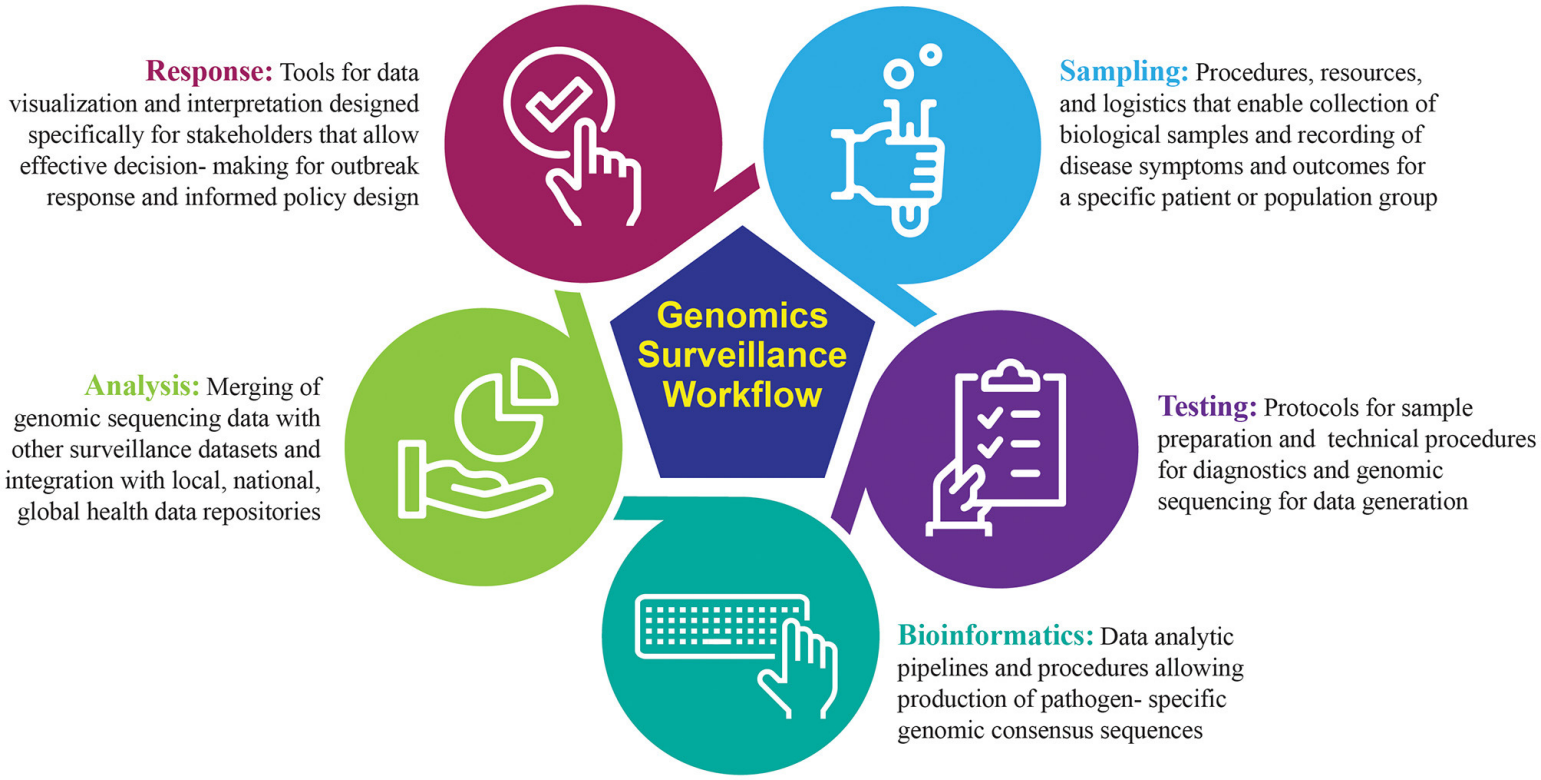
Overview of end-to-end genomics-based surveillance workflow.

Genomic sequencing is a powerful tool for detection and routine surveillance of emerging pathogens. It can provide absolute discriminatory power of viral pathogens during an outbreak by identifying single-point mutations or genes associated with increased virulence and pathogenicity in bacterial genomes. One of the most valuable features of sequencing is culture-free identification of pathogens, decreasing bias and time-to-answer. Real-time sequencing for the purpose of pathogen diagnostics provides critical epidemiological data to understand pathogen transmission, commonly referred to as genomic epidemiology (21). Broadly, the genomic surveillance workflow can be segmented into five distinct modules: sampling, testing, bioinformatics, analysis, and response (Figure 1). The success of a genomic surveillance program requires that necessary data are generated at the appropriate scale and can be interpreted within a relevant timeframe to support decision making; for example, early detection of an emerging infectious disease requires active sequencing capacity at sufficient scale in high vulnerability areas, while diagnostic sequencing requires actionable results in as close to real-time as possible (22).

**Figure 2:**
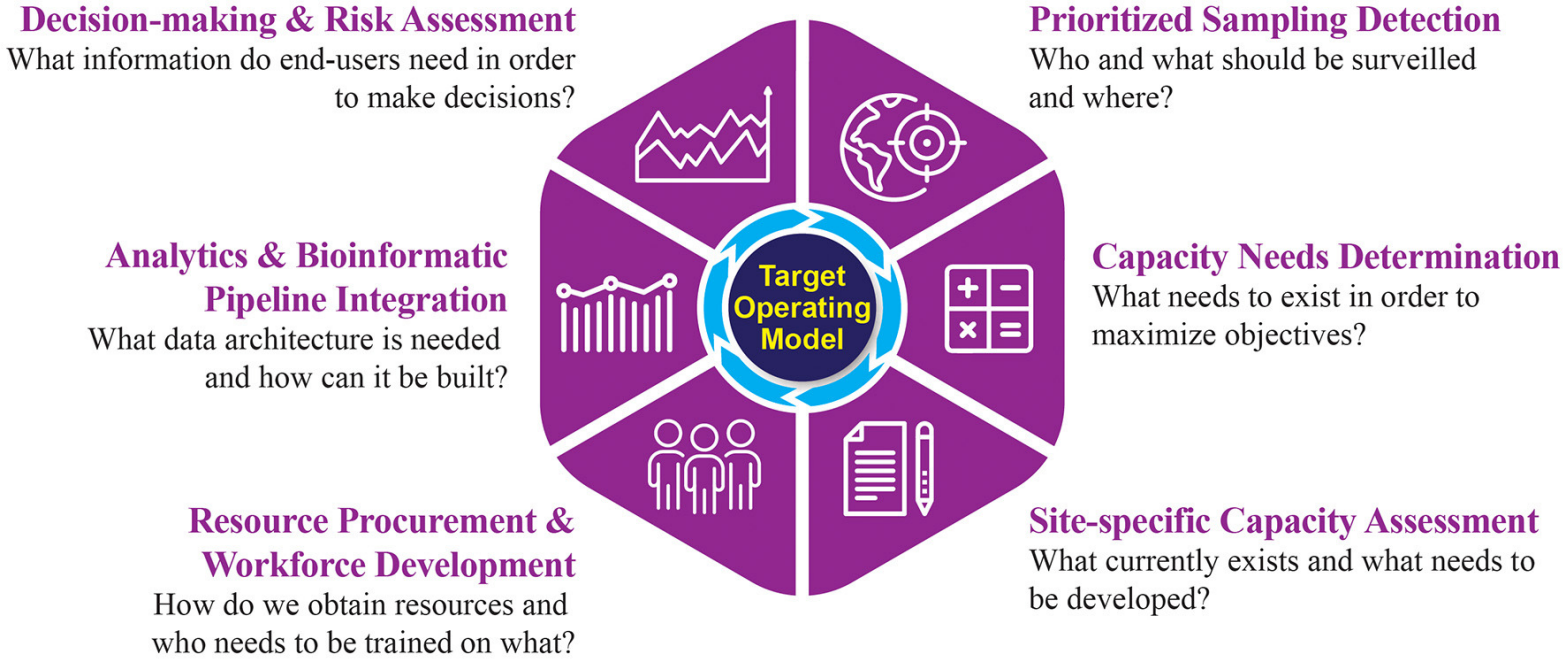
A target operating model for the implementation of genomics-driven surveillance activities.

A clear mechanism to define genomic surveillance objectives, develop an implementation strategy to reach them, and evaluate program success has been lacking. The TOM described here is a tool to define an implementation strategy for genomic surveillance, fit to both stakeholder objectives and end-user needs. Each element of the TOM captures critical information to inform strategy development and outlines key considerations to achieve programmatic objectives (Figure 2).

### Prioritized Sampling Detection

The first element of the TOM specifies objectives of the genomic surveillance program, including the desired pathogen(s) for detection and the catchment area parameters for establishing a genomic surveillance workflow. This element incorporates anthropological information describing the local population and their environment. Baseline data, such as age distribution and common underlying health conditions, contextualizes downstream epidemiologic analyses. Pathogen-specific information that describes current and historical trends for endemic diseases helps guide the prioritization of pathogens. Effort should be made to acknowledge local practices that increase risk of exposure to emerging infectious disease, such as the presence of live markets or burial customs, as these can be particularly relevant when designing surveillance strategies within a given region. This information can be gathered through a combination of literature reviews, interviews with those who have local expertise, and in partnership with local government.

Compiling this broad scope of data provides essential information to define the target population and catchment area parameters for high-priority pathogens. Together, this information permits further prioritization of surveillance activities and their required technologies. Passive and scalable methods such as satellite imagery of habitat destruction, analyses of regional climate fluctuation, or dynamics of human and animal migration could all provide additional information to further refine an ideal region for surveillance.

### Capacity Needs Determination

The information collected to prioritize sampling is then used as the basis to determine optimal capacity and associated resource costs over the entire genomic surveillance workflow. Calculations to define optimal capacity needs should be guided by historical data, providing estimates of required sampling throughput to achieve the stated objectives. While there are tools that can assist in generating these estimates (23), there is a considerable need to develop tools specifically designed for genomic surveillance programs. Information gathered in TOM element one, such as historical trends and priority pathogens, will be essential in framing these calculations, ideally yielding a cost analysis for implementation and operational phases, including materials, equipment, infrastructure, and labor costs to achieve the desired objectives.

This analysis will further refine the scope and design of the program by assessing feasibility given financial constraints. It is critical to ensure that any planned surveillance programs are sustainable. For example, it may not be feasible to build a program to survey both blood-born and respiratory pathogens as the sampling and downstream processing requirements are different, which can substantially increase costs. However, coupled with the product of element one, this analysis will support further prioritization of objectives and will provide a roadmap for future expansion.

### Site-Specific Capacity Assessment

Once calculations have been completed to determine the optimal needs for deploying a genomic surveillance workflow, the third element outlines an onsite detailed assessment of current capacity. Assessments should be conducted across stakeholders and alongside the local workforce to ensure every module that comprises the genomic sequencing workflow is scrutinized. This includes a detailed analysis of medical and laboratory capabilities as well as biosafety infrastructure, sample transport, record keeping, and data storage. Multiple tools are available to evaluate established surveillance systems to improve efficiency in data generation; however, the majority of these focus on laboratory performance and data quality while undervaluing operational considerations (24, 25). Future efforts should develop readiness and requirement assessment tools specific for the full genomic surveillance workflow.

Capacity assessments should also include neighboring regions to identify opportunities for collaboration and resource sharing. This process is often overlooked in surveillance program development, leading to siloed systems that would benefit from an information exchange but are not interoperable. There may be no need to build out every component of the genomic sequencing workflow locally if collaboration is available. For example, in development of a new program, it may be more cost-efficient while maintaining time requirements to leverage existing cold-chain delivery mechanisms that allow for rapid delivery of the locally collected samples to a neighboring locale for diagnostics or sequencing capabilities. The outcome of this TOM element is a streamlined workflow facilitating inclusive sampling of the defined population, rapid diagnosis and sample delivery, efficient genomic sequencing, swift downstream analysis, and delivery of results.

### Resource Procurement and Workforce Development

This element defines procurement and training processes required to implement the workflow. Resource and staffing limitations must be addressed for initiatives to be successful and achieve desired outcomes. As assessed in TOM element two, there are costs associated with implementation and operations. These costs manifest not just monetarily, but also in areas such as time needed to transport samples and obtain reagents, staff onboarding, and continued availability of information technology and electrical infrastructure (26).

The local workforce needs to be trained and empowered to sustain local capability. Building out local expertise is crucial, but the breadth of required knowledge across biology, bioinformatics, and information technology is much too broad for a single individual. Hiring and cross-training staff to fill one or more required domains is often necessary to facilitate implementation of a workflow, and twinning partnerships may facilitate external support, particularly for nascent programs (27). Ongoing training and outreach are required to ensure a skilled labor pool and continuous independent operation.

Equipment, infrastructure, software, reagents, and other assets need to be procured to facilitate ready adoption; selections should prioritize usability and accessibility of training. These capabilities should either be tailored to available expertise or incorporate training to be not only administered during the implementation process but also continually available during operation. Technologies and processes that require highly specialized expertise or are incongruent with existing workflows and priorities will be abandoned in favor of older, more familiar methods. Data architectures must be planned with appropriate data storage, internet connectivity, processing power, and bandwidth in mind at all incorporated sites to allow timely, near-autonomous transport of data and information. Considerations should include not only hardware and software, but also a critical awareness of their geographic locations, facilities, and proximity to reliable utilities to avoid the implementation of a brittle capacity (28).

### Analytics and Bioinformatics Pipeline Integration

Once the means to develop or obtain assets are resolved, data architectures must be leveraged to enable connectivity of data systems and enable data analysis to support the goals of the workflow. A central site needs to be identified and all data pipelines must terminate there. Pipelines should be established such that they are resilient and fault-tolerant to internet and power outages. Data standards must be evaluated to achieve the delicate balance of sufficient structure to ensure high quality data without hindering or preventing data reporting and submission. Establishing consistent data formats across sites enables reliable processing and quality checking of data standards compliance. Where possible, the minimum amount of data to support analyses should be transferred to meet timeliness objectives and overcome bandwidth and storage limitations. Data pipelines should incorporate a transformation step that directly integrates the data with an existing surveillance system or produces a visual artifact.

Mechanisms by which sequence data or its metadata will be transported to a central surveillance system and corresponding repositories needs to be determined. A plethora of software tools and libraries exist which enable processing of sequence data. More importantly, workflow management capabilities that facilitate modular development and integration of analytic pipelines have recently become more widely available (29). Selecting analytic pipelines that both reduce complexity of implementation and lower required technical expertise for execution and interpretation is critical.

### Decision-Making and Risk Assessment

The final element aims to isolate the information required by end users to enable them to make specific decisions and implement critical response measures. Using artifacts produced in the previous TOM element, analysts can begin to interpret the data. It is imperative to decide how epidemiological and genomic data are integrated and presented for end-user consumption; however, the specific approach will vary based on the program objectives. Just as data fidelity and timeliness is important to the end-user, so is the ability to convey information in a readily interpretable manner. For example, a geographical categorization of data is appropriate to evaluate the prevalence of pathogens stratified by variants across regions, while a network graph indicates transmission chains by utilizing case and genomic data. Determining the correct visualization for the right audience such that there is little ambiguity is critical. Inclusion of techniques such as statistical anomaly detection and forecasting should be considered but care must be taken to ensure that outputs of these elements are overlaid and communicated appropriately. Regardless of the method used, information outputs should be easily configurable, interpretable, and shareable to ease the analytic burden on end-users and place an emphasis on making decisions.

## Discussion

The importance of genomics-based surveillance has been clearly demonstrated in diverse contexts, including characterizing pathogen transmission routes, contact tracing, vaccine design, and more. The COVID-19 pandemic has reinforced the need for genomic surveillance and integration of both genomic and epidemiological data to support stronger decision making in response to emerging infectious disease. Additionally, uniting genomic surveillance in human and animal populations can provide predictive indicators of potential or emerging outbreaks. As such, genomic surveillance represents a critical capability in preparedness and response.

To fully realize the promise of genomic surveillance, several key requirements must be met. First, genomic surveillance data must be meaningfully integrated with traditional health surveillance data for clinical characterization of an emerging pathogen. Second, sufficient sampling coverage and throughput is necessary to adequately support research and an informed public health response. Third, genomic surveillance infrastructure must be present and operational, featuring sufficient flexibility to support broad-spectrum pathogen surveillance in order to be relevant in emerging outbreak scenarios. Finally, genomic surveillance initiatives must be sustainable, providing sufficient value to local and national decision makers to justify the investment required for continuing operation, maintenance, and improvement.

The genomics-based surveillance TOM presented here provides an in-depth framework for development of an implementation strategy for new genomic surveillance programs. While relevant in both high-income and LMIC contexts, we have focused on an implementation scenario in LMICs given observed challenges in sustainability of similar programs. Additionally, the increased burden of infectious disease on LMICs and frequently insufficient infrastructure and allocation of resources for preparedness and response capabilities mandates deeply cost-effective surveillance strategies. As such, the TOM provides a mechanism to ensure surveillance objectives established by a given LMIC are clearly presented and upheld in the development of an implementation strategy. Use of the TOM to evaluate and adapt or refine an existing genomic surveillance program will also ensure minimal investments achieve the greatest possible impact.

The TOM provides a flexible framework upon which specific priorities for sampling and capacity development can be defined based on a combinatorial evaluation of surveillance priorities, available resources, and stakeholder needs. This operational model can be used to drive development and refinement of surveillance programs through top-down planning and allows gap identification within any given surveillance workflow. Integrating interdisciplinary models to support TOM assessments will provide a rich quantitative basis for prioritization of resource allocation in development or adaptation of genomic surveillance programs. As new global health capabilities are developed and refined, the TOM is understood to be an evolving product, providing a reflection of both global and national health priorities and continually strengthened by advances in knowledge surrounding health and infectious disease. As technical and analytic resources are identified, a common resource repository, including trainings and lessons learned regarding genomic surveillance implementation, can be developed. Further adaptation of the TOM to broader health surveillance objectives (e.g., pathogen emergence, livestock surveillance, and more) should be explored as well.

We have presented here a target operating model for the establishment of a genomic surveillance program for infectious disease in LMICs; however, we recognize additional needs in the sustainment of newly developed capacity in COVID-19 response in high-income countries as well. The logic and decision framework underlying the LMIC TOM instantiation is easily translated to highly-resourced settings. Application of the TOM to assess genomic surveillance program performance and opportunities for refinement or realignment against national surveillance priorities for any nation should be built into existing program reviews to ensure our health surveillance capabilities remain strong and vibrant to protect all nations from the next emerging health crisis.

## Data Availability Statement

The original contributions presented in the study are included in the article, further inquiries can be directed to the corresponding author.

## Author Contributions

NL wrote the majority of the manuscript. NL and MS contributed equally to the concept described in the manuscript. JD, MS, and SL contributed to the content of the manuscript. All authors approved the final version of the manuscript.

## Conflict of Interest

The authors declare that the research was conducted in the absence of any commercial or financial relationships that could be construed as a potential conflict of interest.

## Publisher's Note

All claims expressed in this article are solely those of the authors and do not necessarily represent those of their affiliated organizations, or those of the publisher, the editors and the reviewers. Any product that may be evaluated in this article, or claim that may be made by its manufacturer, is not guaranteed or endorsed by the publisher.
